# Cellular Signaling and Anti-Apoptotic Effects of Prolactin-Releasing Peptide and Its Analog on SH-SY5Y Cells

**DOI:** 10.3390/ijms21176343

**Published:** 2020-09-01

**Authors:** Anna Zmeškalová, Andrea Popelová, Aneta Exnerová, Blanka Železná, Jaroslav Kuneš, Lenka Maletínská

**Affiliations:** 1 Institute of Organic Chemistry and Biochemistry, AS CR, Prague 16000, Czech Republic; zmeskalova@uochb.cas.cz (A.Z.); andrea.popelova@uochb.cas.cz (A.P.); aneta.exnerova@uochb.cas.cz (A.E.); zelezna@uochb.cas.cz (B.Z.); kunes@biomed.cas.cz (J.K.); 2 First Faculty of Medicine, Charles University, Prague 12108, Czech Republic; 3 Institute of Physiology, AS CR, Prague 14200, Czech Republic

**Keywords:** prolactin-releasing peptide, cellular signaling, inhibitors, methylglyoxal, neuroprotection, SH-SY5Y, primary neuronal culture

## Abstract

Prolactin-releasing peptide (PrRP), a natural ligand for the GPR10 receptor, is a neuropeptide with anorexigenic and antidiabetic properties. Due to its role in the regulation of food intake, PrRP is a potential drug for obesity treatment and associated type 2 diabetes mellitus (T2DM). Recently, the neuroprotective effects of lipidized PrRP analogs have been proven. In this study, we focused on the molecular mechanisms of action of natural PrRP31 and its lipidized analog palm^11^-PrRP31 in the human neuroblastoma cell line SH-SY5Y to describe their cellular signaling and possible anti-apoptotic properties. PrRP31 significantly upregulated the phosphoinositide-3 kinase-protein kinase B/Akt (PI3K-PKB/Akt) and extracellular signal-regulated kinase/cAMP response element-binding protein (ERK-CREB) signaling pathways that promote metabolic cell survival and growth. In addition, we proved via protein kinase inhibitors that activation of signaling pathways is mediated specifically by PrRP31 and its palmitoylated analog. Furthermore, the potential neuroprotective properties were studied through activation of anti-apoptotic pathways of PrRP31 and palm^11^-PrRP31 using the SH-SY5Y cell line and rat primary neuronal culture stressed with toxic methylglyoxal (MG). The results indicate increased viability of the cells treated with PrRP and palm^11^-PrRP31 and a reduced degree of apoptosis induced by MG, suggesting their potential use in the treatment of neurological disorders.

## 1. Introduction

Neuropeptide with the misleading name prolactin-releasing peptide (PrRP) belongs to the family of RF-amide peptides. PrRP immunoreactive fibers, as well as its receptor GPR10, can be found in the brain in nuclei implicated in food intake and energy balance regulation [1], such as in the nucleus of the solitary tract (NTS) of the brainstem, and in several hypothalamic nuclei, where it acts as an anorexigenic compound [2,3]. Natural PrRP has two equally active isoforms with different lengths: the shorter PrRP20 comprises 20 amino acids, and the longer PrRP31 comprises 31 amino acids [4]. None of the isoforms can decrease food intake after acute peripheral administration [5]. However, our group designed and synthesized a series of lipidized PrRP31 and PrRP20 analogs that are able to act centrally after peripheral administration, as shown by decreased acute food intake and c-Fos activation, namely, in the paraventricular nucleus of the hypothalamus (PVN) and NTS, in fasted C57BL/6 mice [6]. PrRP31 palmitoylated at the N-terminus (palm^1^-PrRP31) or through the γ-Glu-linker at Lys^11^ (instead of natural Arg^11^, palm^11^-PrRP31) were chosen as the most potent analogs, with strong specific binding to the GPR10 receptor and increased stability and bioavailability in blood plasma. Subsequently, in several rodent models of obesity, long-term treatment with palm^1^-PrRP31 or palm^11^-PrRP31 significantly decreased body weight [6,7,8]. Moreover, palm^11^-PrRP31 significantly improved glucose tolerance in Zucker diabetic rats or in spontaneously hypertensive obese rats [7,8].

Antidiabetic and anorexigenic peptides were recently repurposed as potential neuroprotective compounds because type 2 diabetes mellitus (T2DM) and obesity were defined as risk factors increasing the development of Alzheimer’s disease (AD). AD is the most common type of dementia, with a dramatically increasing prevalence associated with memory impairment and progressive cognitive decline. AD is characterized by extracellular senile β-amyloid (Aβ) deposits [9] and intracellular neurofibrillary tangles (NFT) formed by hyperphosphorylated Tau protein [10]. The mechanism of AD development is not well understood; however, one of the hypotheses calls AD type 3 diabetes mellitus because of several features common for both AD and T2DM [11]: insulin resistance, impaired glucose metabolism, increased inflammation or oxidative stress [12,13]. In concordance, impaired insulin signaling leading to hyperphosphorylation of Tau protein was observed in AD patients with T2DM [14]. To prove the potential neuroprotective properties of stable palmitoylated PrRP analogs towards Aβ as well as Tau protein, different mouse models of AD-like pathology were treated with palm^1^-PrRP31 or palm^11^-PrRP31. Palm^1^-PrRP31 ameliorated hippocampal insulin signaling pathways in mice with monosodium glutamate (MSG)-induced obesity and further attenuated Tau hyperphosphorylation at several epitopes [15]. Attenuation of Tau hyperphosphorylation was also observed in the THY-Tau22 transgenic mouse model of AD-like tauopathy [16], where palm^11^-PrRP31 treatment improved short-term spatial memory in the Y-maze test [17]. Furthermore, palm^11^-PrRP31 showed significant beneficial neuroprotective effects in the APP_swe_/PS1_dE9_ mouse model of AD-like Aβ pathology. The peptide reduced amyloid plaque deposition, neuroinflammation, and Tau phosphorylation [18].

While the neuroprotective effect of PrRP analogs has been proven, their mechanism of action is poorly understood. Previously, several in vitro studies proved that PrRP and its analogs activate mitogen-activated protein kinase/extracellular signal-regulated kinase 1/2 (MAPK/ERK1/2) and cAMP response element-binding protein (CREB) pathways, which are important, e.g., for memory formation [19,20]. An in vitro study using the rat cell line GH3 and human PrRP [21] and a later in vivo study on MSG mice treated with palm^1^-PrRP revealed increased activation of kinases implicated in the insulin signaling pathway, such as phosphoinositide-dependent protein kinase-1 (PDK1), Akt, and glycogen synthase kinase 3β (GSK-3β) [15]. Akt is an important anti-apoptotic factor. Decreased Akt activation leads to increased activation of pro-apoptotic proteins, such as Bcl-2-associated X (Bax) or Bcl-2-associated death promoter (Bad) [22]. Moreover, Akt further phosphorylates GSK-3β at Ser9; the increased phosphorylation prevents its kinase activity towards Tau protein [23]. Both of these signaling pathways are activated by insulin, which is important not only in the periphery for glucose uptake but also in the central nervous system. Insulin receptors (IRs) are present in brain regions connected with memory formation and learning, namely, in the cortex and hippocampus [24]. Activation of IR was observed after spatial memory testing in the rat hippocampal CA1 region, suggesting the role of insulin in memory processing [25].

An appropriate in vitro system for studying the neurodegenerative processes connected to T2DM in neurons is treatment with toxic methylglyoxal [1]. MG is a highly reactive product of mainly glucose metabolism that accumulates in a chronic hyperglycemic state, such as T2DM [26]. MG is a neurotoxic mediator of oxidative stress in the progression of AD and is capable of activating many redox signaling pathways, leading to increased apoptosis through increased activation of pro-apoptotic Bax and decreased activation of anti-apoptotic Bcl-2 [27]. Moreover, MG induces Tau hyperphosphorylation by enhancing kinase activities and reducing phosphatase levels [28].

In the present study, we aimed to identify the signaling pathways activated by PrRP with an emphasis on pathways implicated in anti-apoptotic actions using the human neuroblastoma cell line SH-SY5Y, which is widely used for the study of neurodegeneration, as well as rat primary cortical neurons. Further, we assessed the effects of PrRP and its palmitoylated analog palm^11^-PrRP31 in the prevention of neurotoxic effects of MG and after exposure to MG, which induced decreased cell viability and activation of pro-apoptotic enzymes.

## 2. Results

### 2.1. PrRP31 and Palm^11^-PrRP31 Activated the PI3K/Akt Signaling Pathway in SH-SY5Y Cells

**Figure 1:**
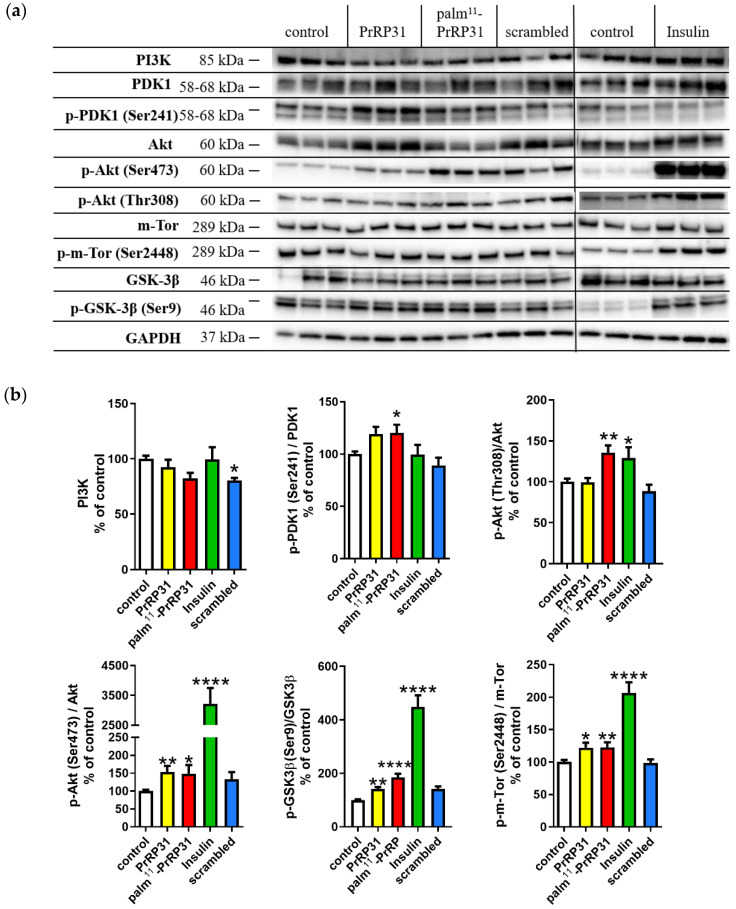
Prolactin-releasing peptide 31 (PrRP31) and palm^11^-PrRP31 increased phosphoinositide-3 kinase (PI3K/Akt) signaling pathway in SH-SY5Y cells. Immunoblots: (**a**) and their quantifications: (**b**). SH-SY5Y cells were incubated either with PrRP31 and its palmitoylated analog and scrambled at a final concentration 1 × 10^-5^ M, or with insulin at the final concentration 1 × 10^−7^ M at 37 °C for 8 min, or medium alone (control). Activation was expressed as the ratio of the activated protein to the total amount of the protein, both normalized to loading control, glyceraldehyde 3-phosphate dehydrogenase (GAPDH). Data are presented as the means ± SEM as a percentage of control cells analyzed by one-way ANOVA followed by the Dunnett post hoc test, * *p* < 0.5, ** *p* < 0.01, **** *p* < 0.0001 (*n* = 3, each sample in triplicates).

The presence of PrRP receptors in SH-SY5Y cells was confirmed by specific binding of ^125^I-PrRP31. Compared to total ^125^I-PrRP31 binding, the nonspecific binding to SH-SY5Y cells was lower than 15%. Therefore, we can assume specific binding sites for PrRP and its lipidized analog, thus, subsequently, activation of prospective signaling pathways via PrRP receptors. PrRP31 and palm^11^-PrRP31 significantly increased the level of p-Akt (Ser473)/Akt by 53% and 48%, respectively. Palm^11^-PrRP31 also significantly increased the level of p-Akt (Thr308)/Akt by 36% (Figure 1). PrRP31 and palm^11^-PrRP31 also significantly increased levels of the p-GSK3β (inhibitory Ser9)/GSK3β by 41% and 85%, respectively, and the p-m-Tor (Ser2448)/m-Tor both by 22%. palm^11^-PrRP31 increased the level of p-PDK1 (Ser241)/PDK1 by 20% (Figure 1). As expected, insulin as a positive control significantly activated proteins of the insulin signaling cascade, and scrambled-palm^11^-PrRP31 analog (scrambled), as a negative control, did not significantly affect the PI3K/Akt pathway (Figure 1).

### 2.2. PrRP31 and Palm^11^-PrRP31 Activated the ERK-CREB Signaling Pathway in SH-SY5Y Cells

**Figure 2:**
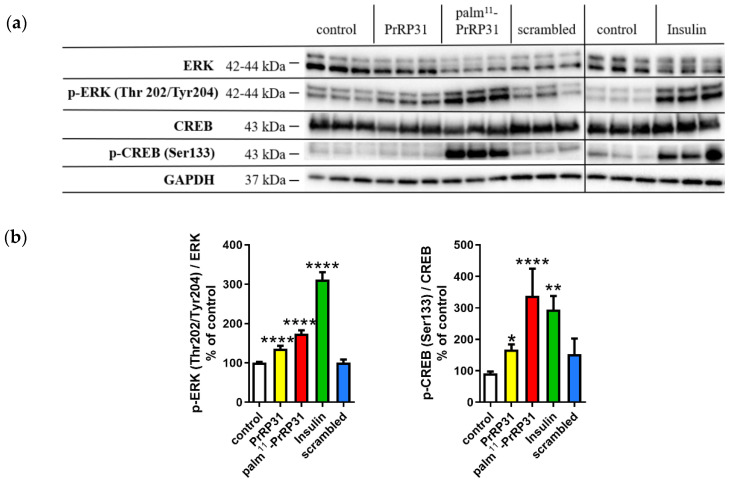
PrRP31 and palm^11^-PrRP31 increased phosphorylation of extracellular signal-regulated kinase/cAMP response element-binding protein (ERK-CREB) signaling pathway in SH-SY5Y cells. Immunoblots: (**a**) and their quantifications: (**b**). SH-SY5Y cells were incubated either with PrRP31 and its palmitoylated analog and scrambled at a final concentration 1 × 10^−5^ M, or with insulin at the final concentration 1 × 10^−7^ M at 37 °C for 8 min, or medium alone. Activation was expressed as the ratio of the activated protein to the total amount of the protein, both normalized to loading control, GAPDH. Data are presented as the means ± SEM as a percentage of control cells treated with medium alone analyzed by one-way ANOVA followed by the Dunnett post hoc test, * *p* < 0.5, ** *p* < 0.01, **** *p* < 0.0001 (*n* = 3, each sample in triplicates).

PrRP31 and palm^11^-PrRP31 significantly increased both p-ERK (Thr 202/Tyr204)/ERK by 36% and 74%, respectively, and p-CREB (Ser133)/CREB by 68% and 238%, respectively. Insulin was used as a positive control, scrambled PrRP31 as negative control. Representative immunoblots and their quantifications are shown in Figure 2.

### 2.3. Inhibitors of Signaling Pathways Proved That Activation of Signaling Pathways Is Mediated Specifically via PrRP31 and Its Palmitoylated Analog

**Figure 3:**
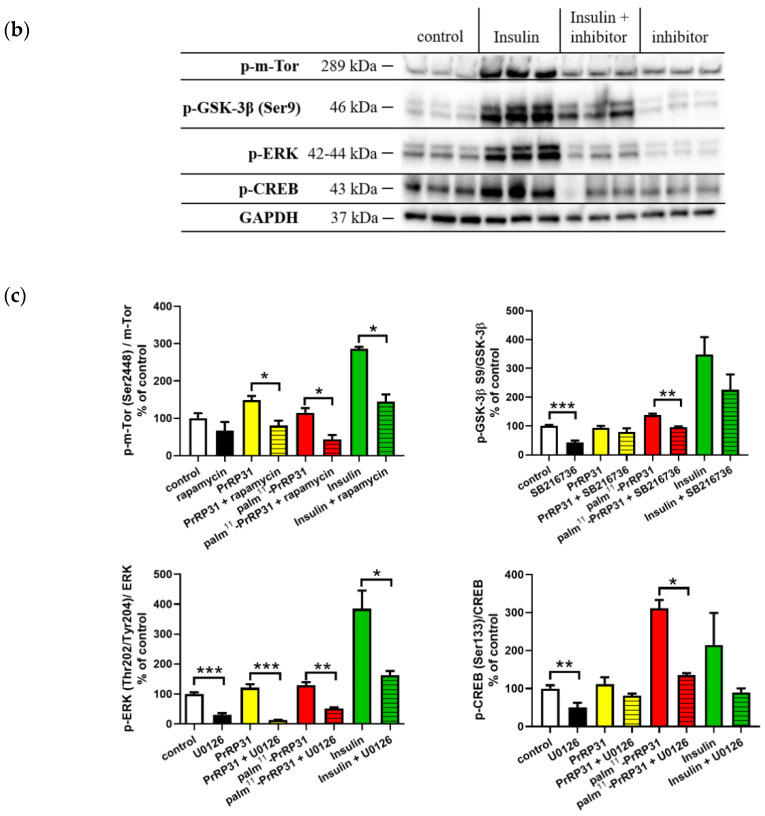
Inhibitors proved that activation of signaling pathways were mediated exclusively via PrRP31 and its palmitoylated analog. Immunoblots with PrRP31 and palm^11^-PrRP31: (**a**) and insulin: (**b**) and their quantifications: (**c**). SH-SY5Y cells were incubated firstly with selective inhibitors for 2 min and then either with PrRP31 and its palmitoylated analog at a final concentration 1 × 10^−5^ M, or with insulin in the final concentration 1 × 10^−7^ M at 37 °C for 8 min, or alone in serum-free medium. Activated protein was normalized to loading control, GAPDH. Data are presented as the means ± SEM as a percentage of control cells treated with medium alone. Analysis is made between groups as shown in the graph by a Student’s *t*-test, * *p* < 0.5, ** *p* < 0.01, *** *p* < 0.001(each sample in triplicates).

From the results shown in Figure 3, it is evident that specific inhibitors significantly decreased the phosphorylation of relevant signaling proteins in the majority of experiments. After the application of PrRP31, palm^11^-PrRP31, and insulin, p-m-Tor (Ser2448) was significantly increased; this increase was inhibited by treatment with rapamycin, a selective inhibitor of m-TorC1 complex under the control level. A slight increase was also observed in p-GSK-3β (Ser9) after incubation with palm^11^-PrRP31 and insulin. A selective inhibitor of GSK-3β, SB216736, significantly reversed the palm^11^-PrRP31 effect and tended to decrease the level in the cells treated with insulin. p-ERK and p-CREB were increased after incubation with all three analogs. This effect was significantly blocked under the control level, by a selective inhibitor of MAPK kinase activity, U0126.

### 2.4. Methylglyoxal-Induced Cytotoxicity

**Figure 4:**
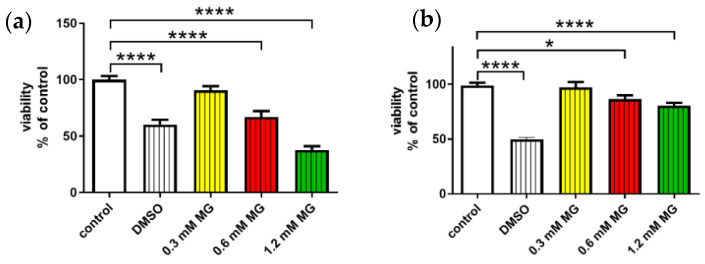
Optimization of methylglyoxal [1] concentration for measuring potential anti-apoptotic properties of PrRP. MG toxicity was measured with an MTT assay: (**a**) in SH-SY5Y cells; (**b**) in rat primary neuronal culture. Data are presented as the means ± SEM as a percentage of control cells treated with vehicle. Statistics: one-way ANOVA followed by the Dunnett post hoc test, * *p* < 0.5, **** *p* < 0.0001 (*n* = 3, each sample in octuplicates).

For optimization, MG at concentrations of 0.3 mM, 0.6 mM and 1.2 mM was used. DMSO, known for its cytotoxic properties, was used as a comparator of MG-induced cytotoxicity. MG at concentrations of 0.6 mM and 1.2 mM significantly decreased cell viability by 33% and 62%, respectively in SH-SY5Y cell line and by 14% and 20%, respectively, in rat primary cortical culture, compared to that of the control cells and was, therefore, used in subsequent experiments (Figure 4).

#### 2.4.1. PrRP31 and Its Palmitoylated Analog Protected SH-SY5Y Cells from the Cytotoxic Effects of MG

**Figure 5:**
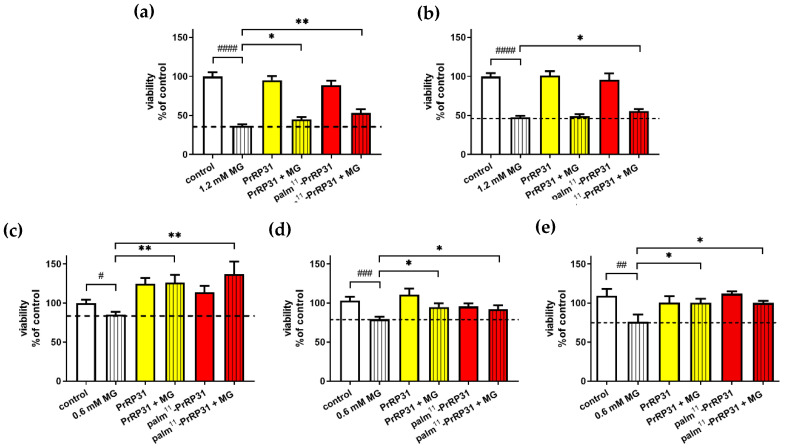
PrRP31 and its analog increased cell viability during the MG-induced stress. Pre-treatment for 4 h with PrRP31 and palm^11^-PrRP31 at a concentration of: (**a**) 1 × 10^−5^ M; or (**b**) 1 × 10^−7^ M, followed by exposure to 1.2 mM MG for 16 h in SH-SY5Y cells. Pre-treatment for 4 h with PrRP31 and palm^11^-PrRP31 at a concentration of: (**c**) 1 × 10^−5^ M; (**d**) 1 × 10^−7^ M in SH-SY5Y cells; or (**e**) 1 × 10^−5^ M in rat primary neuronal culture, followed by exposure to 0.6 mM MG for 16 h in SH-SY5Y cells. MG toxicity was measured with MTT assay. Data are presented as the means ± SEM as a percentage of control cells treated with vehicle analyzed by a Student’s *t*-test vs. 0.6 mM MG # *p* < 0.5, ## *p* < 0.01, ### *p* < 0.001, #### *p* < 0.0001 (*n* = 3, each sample in octuplicates). Control vs. 0.6 mM MG analyzed by a Student´s *t*-test, * *p* < 0.5, ** *p* < 0.01.

MG at a concentration of 1.2 mM significantly decreased the cell viability compared to that of the control cells in the MTT assay. Four-hour pretreatment with PrRP31 and its palmitoylated analog at a concentration of 1 × 10^−5^ M in SH-SY5Y cells resulted in increased cell viability to 45% and 53 %, respectively, compared to 37% of the non-pretreated cells administered with 1.2 mM MG (Figure 5).

Only pretreatment with palm^11^-PrRP31 increased viability to 56%, but not PrRP31 at 1 × 10^−7^ M compared to 48% of the non-pretreated cells treated with 1.2 mM MG only (Figure 5). MG at a concentration of 0.6 mM also significantly decreased cell viability compared to that of the control cells in the MTT assay. Pretreatment with both PrRP31 and its palmitoylated analog at a concentration of 1 × 10^−5^ M resulted in increased cell viability to 126% and 137%, respectively, compared to 85% of the non-pretreated cells treated with 0.6 mM MG only. Pretreatment at a concentration of 1 × 10^−7^ M in SH-SY5Y cells resulted in increased cell viability to 95% and 92%, respectively, compared to 79% of the non-pretreated cells treated with 0.6 mM MG only (Figure 5).

Pretreatment with PrRP31 and palm^11^-PrRP31 at a concentration of 1 × 10^−5^ M in rat primary neuronal culture resulted in enhanced cell viability to 100% compared to 76% of the non-pretreated cells administered with 0.6 mM MG (Figure 5).

#### 2.4.2. PrRP and Its Palmitoylated Analog Increased the Viability of SH-SY5Y Cells after 16 h of Exposure to MG

**Figure 6:**
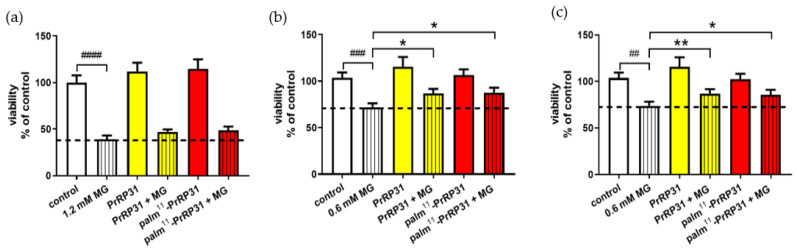
PrRP31 increased cell viability of SH-SY5Y cells after 16-h exposure to MG. Treatment for 4 h with PrRP31 and palm^11^-PrRP31 at a concentration of: (**a**) 1 × 10^−5^ M; after exposure to 1.2 mM MG for 16 h in SH-SY5Y cells. Treatment for 4 h with PrRP31 and palm^11^-PrRP31 at a concentration of: (**b**) 1 × 10^−5^ M; (**c**) 1 × 10^−7^ M, after exposure to 0.6 mM MG for 16 h in SH-SY5Y cells. MG toxicity measured with MTT assay. Data are presented as the means ± SEM as a percentage of control cells treated with vehicle analyzed by a Student´s *t*-test vs. 0.6 mM MG ## *p* < 0.01, ### *p* < 0.001, #### *p* < 0.0001 (*n* = 3, each sample in octuplicates). Control vs. 0.6 mM MG analyzed by a Student´s *t*-test, * *p* < 0.5, ** *p* < 0.01.

MG at a concentration of 1.2 mM again significantly decreased cell viability to 39% compared to the control cells. Four-hour treatment with PrRP31 and its palmitoylated analog at a concentration of 1 × 10^−5^ M in SH-SY5Y cells resulted in a nonsignificant increase in cell viability compared to that of the cells administered with 1.2 mM MG (Figure 6).

MG at a concentration of 0.6 mM also significantly decreased cell viability compared to that of the control cells in the MTT test. Treatment with PrRP31 and palm^11^-PrRP31 at a concentration of 1 × 10^−5^ M in SH-SY5Y cells resulted in increased cell viability to 87% compared to 72% of the nontreated cells treated with 0.6 mM MG (Figure 6). Treatment with PrRP31 and palm^11^-PrRP31 at a concentration of 1 × 10^−7^ M resulted in increased cell viability to 87% compared to 74% of the nontreated cells treated with 0.6 mM MG (Figure 6).

### 2.5. PrRP31 and Palm^11^-PrRP31 Induced Anti-Apoptotic Signaling in SH-SY5Y Cells

**Figure 7:**
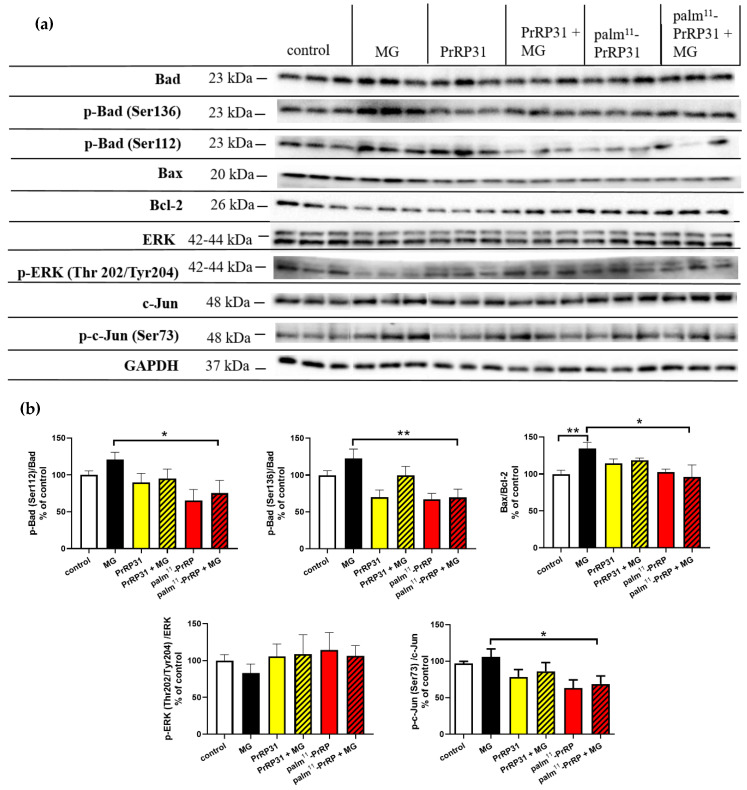
PrRP31 and palm^11^-PrRP31 affected anti-apoptotic signaling in SH-SY5Y cells. Immunoblots: (**a**) and their quantifications: (**b**). SH-SY5Y cells were pretreated with PrRP31 and palm^11^-PrRP31 at a concentration of 1 × 10^−5^ M for 4 h and then stressed with 0.6 mM MG for 16 h. Activation was expressed as the ratio of the activated protein to the total amount of the protein, both normalized to loading control, GAPDH. Data are presented as the means ± SEM as a percentage of control cells treated with medium alone. Analysis is made vs. MG by a Student’s *t*-test, * *p* < 0.5, ** *p* < 0.01 (each sample in triplicates).

Representative immunoblots of SH-SY5Y cells pretreated with the peptides and then stressed with MG and their quantification are shown in Figure 7. MG increased the level of p-c-Jun (Ser73)/c-Jun, which was significantly decreased by 37% in the SH-SY5Y cells pretreated with palm^11^-PrRP31. PrRP31 tended to decrease p-c-Jun. The level of p-ERK (Thr202/Tyr204)/ERK was affected neither by MG nor with PrRP31 and palm^11^-PrRP31 pretreatment. The levels of p-Bad (Ser112)/Bad and p-Bad (Ser136)/Bad were increased after MG, tended to decrease in the cells pretreated with PrRP31, and were significantly decreased by 46% and 52%, respectively, in the cells pretreated with palm^11^-PrRP31. The ratio of Bax/Bcl−2 significantly increased after MG exposure was attenuated significantly by 38% in the cells pretreated with palm^11^-PrRP31.

### 2.6. PrRP31 and Palm^11^-PrRP31 Attenuated Tau Hyperphosphorylation at Different Epitopes in SH-SY5Y Cells

**Figure 8:**
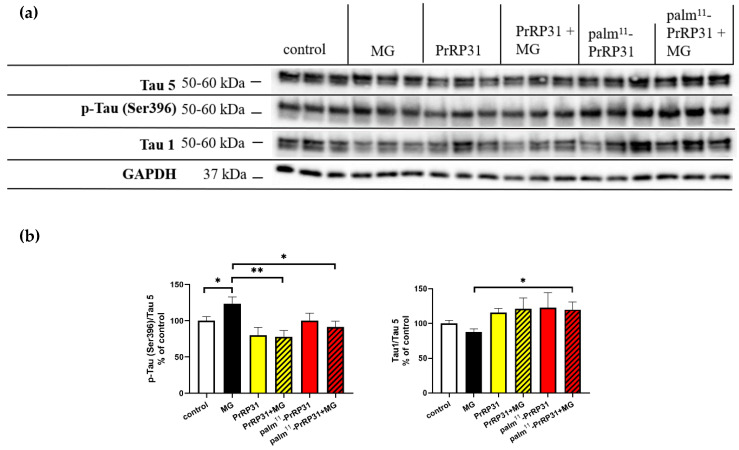
PrRP31 and palm^11^-PrRP31 attenuate Tau hyperphosphorylation at the epitope Ser396 in SH-SY5Y. Immunoblots with: (**a**) and their quantifications: (**b**). SH-SY5Y cells were pretreated with PrRP31 and palm^11^-PrRP31 at a concentration of 1 × 10^−5^ M for 4 h and then stressed with 0.6 mM MG for 16 h. Activation was expressed as the ratio of the activated protein to the total amount of the protein, both normalized to loading control, GAPDH. Data are presented as the means ± SEM as a percentage of control cells treated with medium alone. Analysis is made vs. MG by a Student’s *t*-test, * *p* < 0.5, ** *p* < 0.01 (each sample replicate in triplicate).

MG increased Tau phosphorylation at Ser396 (Figure 8). Pretreatment with either PrRP31 or palm^11^-PrRP31 significantly attenuated this phosphorylation by 46% and 32%, respectively. The levels of Tau phosphorylation at Ser198/199/202 (Tau 1)/Tau 5 tended to increase after MG, as indicated by the decreased levels of the Tau 1 antibody that detects unphosphorylated Tau at the mentioned epitopes. Pretreatment with PrRP31 tended to decrease the phosphorylation induced by MG, as indicated by the Tau 1 antibody, while palm^11^-PrRP31 significantly attenuated Tau phosphorylation shown by an increase in unphosphorylated Tau by 32%.

## 3. Discussion

Obesity and related comorbidities, especially neurological disorders, are dramatically increasing in prevalence worldwide, but effective treatments are lacking. Anorexigenic peptides may be potentially powerful agents for their treatment; however, their mechanism of action is poorly understood. In this study, we investigated the cell signaling of the anorexigenic neuropeptide PrRP31 and its lipidized analog palm^11^-PrRP31 together with insulin whose neuroprotective properties has been already proven [29]. We also studied the potential neuroprotective and anti-apoptotic effects of these peptides in two neuronal models stressed with neurototoxic MG: the human neuroblastoma cell line SH-SY5Y and cortical neurons from rat primary neuronal cultures.

**Figure 9:**
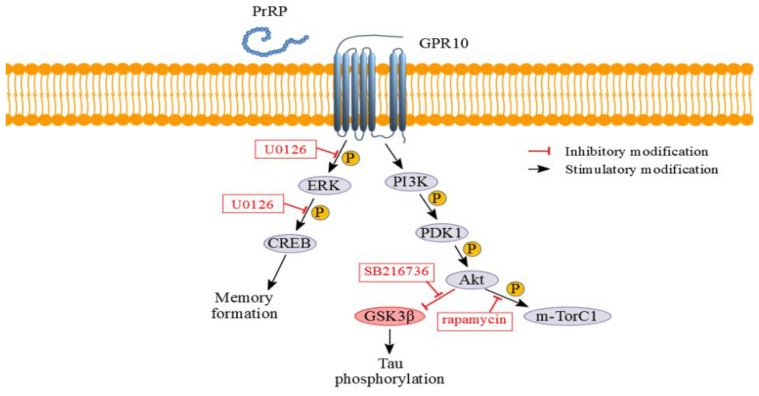
Schematic view of PrRP signaling pathways; P–phosphorylation.

Tracking of cell signaling pathways can help elucidate the molecular mechanism underlying a potent neuroprotective effect of PrRP (Figure 9). From our previous in vitro [30] and in vivo studies [31,32], PI3K/Akt and ERK-CREB signaling pathways are known to be activated by PrRP and its analogs. In this study, incubation of the SH-SY5Y cell line with PrRP31 and palm^11^-PrRP31 increased the activation of proteins that are part of the insulin signaling pathway, namely PDK1 and Akt kinases, similarly as insulin itself in the study of Varghese et al. [33]. In the brain, through PI3K/Akt signaling pathway insulin may act as a neuromodulator, influencing the release and reuptake of neurotransmitters [34], neuronal survival [35,36], synapse formation and plasticity [37,38,39], and learning and memory [25]; hence, insulin was chosen as a positive control [15,40,41]. Impaired activation of this pathway is often linked to neurodegenerative development. In this study, it is proven that PrRP and its lipidized analog participate in the activation of this signaling pathway; therefore, it could be linked to the possible neuroprotective effect of PrRP31 [42].

Furthermore, PrRP31 and its analog significantly increased the phosphorylation of m-Tor, which belongs to the PI3K-related kinase protein family [43,44]. m-Tor may exist as m-Tor complex 1 (m-TorC1) or m-Tor complex 2 (m-TorC2). The key component of m-TorC2 is a rapamycin-insensitive companion of mammalian target of rapamycin (RICTOR), which, unlike m-TorC1, is not directly inhibited by rapamycin. In our study, rapamycin reduced the activation induced by PrRP31 and palm^11^-PrRP31. Therefore, we assume that PrRP31 and palm^11^-PrRP31 increased the activation of m-TorC1, which is involved in the molecular mechanism of dendritic branching and is important for the enhancement of synaptic transmission [45]. m-Tor plays an important role in the growth and differentiation of neural tissue, and its decreased activation can lead to memory impairment and inhibition of the ability to learn [46].

Another kinase implicated in the regulation of cellular metabolism is GSK-3β. In our study, PrRP31 and palm^11^-PrRP31 increased phosphorylation of GSK-3β at Ser9, indicating inhibition of kinase activity of GSK-3β towards Tau protein, manifested by decreased phosphorylation of Tau protein, similarly as in our previous study with MSG mice [15]. Therefore, inhibition of GSK-3β can have a potential neuroprotective effect acting against pathological hyperphosphorylation of Tau protein [47,48,49]. A selective inhibitor of GSK-3β, SB216736, confirmed that PrRP31 and its palmitoylated analog inhibited specifically PrRP-activated phosphorylation of GSK3β at Ser9.

Decreased activation of the ERK-CREB pathway, which physiologically promotes the metabolism of cell survival, growth, cell proliferation, and differentiation [50], can play an important role in the development of neurodegenerative diseases, such as AD [51]. Insulin-induced activation of ERK has been suggested as a crucial player in synaptic and neuronal plasticity [52]. ERK is also an essential component of the signal transduction mechanisms subserving behavioral memory formation [19,25,52,53]. Our results showed that PrRP31 and palm^11^-PrRP31 rapidly increased the activation of ERK and CREB proteins, similar to our and other studies [6,19,20], which again confirmed the potential neuroprotective properties of PrRP31 and its palmitoylated analog, similarly to insulin. U0126, a selective inhibitor of MAPK kinase activity, proved that activation of the ERK-CREB signaling pathway is induced specifically by PrRP and its palmitoylated analog, as the inhibitor attenuated PrRP-mediated phosphorylation of both ERK and CREB.

PrRP and its lipidized analog increased PDK1 and Akt which are part of the insulin signaling pathway, increased activation of mTorC1 and phosphorylation of GSK-3β at Ser9, and furthermore, it increased activation of ERK-CREB pathway in the same manner as insulin, which is required for neuronal synaptic and dendritic plasticity, for learning, and for memory formation. We observed activation of these signaling pathways in our previous in vivo models of neurodegeneration [31,32]; hence these could be the main pathways contributing to the potential neuroprotective effect of PrRP. Therefore, PrRP can have similar positive effects on neuropathological processes as insulin [29], thereby eventually curbing the development and progression of AD [54].

MG, a reactive intermediate of cellular metabolism, is the most potent precursor of advanced glycation end products and is strictly correlated with an increase in oxidative stress in AD [55]. Therefore, MG can be a useful tool for screening potential neuroprotective compounds that ameliorate oxidative stress [56]. Similarly, as in the study with the glucagon-like peptide 1 analog liraglutide, where liraglutide enhanced SH-SY5Y cell viability [57], PrRP31 and palm^11^-PrRP31 increased viability determined by the MTT test in the MG-stressed SH-SY5Y cells.

Pretreatment of SH-SY5Y cells with both PrRP31 and palm^11^-PrRP31 at a concentration of 1 × 10^-5^ M prevented the cytotoxic effects of 1.2 mM MG. At 1 × 10^−7^ M, palm^11^-PrRP31 exhibited significantly increased viability. Lower stability of PrRP than that of the palm^11^-PrRP31 was probably the reason why the deleterious effect of 1.2 mM MG on SH-SY5Y cells was not reversed by this peptide at its lower concentration [6].

Pretreatment with PrRP31 and palm^11^-PrRP31 at concentrations of 1 × 10^−5^ M and 1 × 10^−7^ M not only significantly prevented the cytotoxic effects of 0.6 mM MG in SH-SY5Y cells but also enhanced viability of rat primary cortical cells affected with 0.6 mM MG.

**Figure 10:**
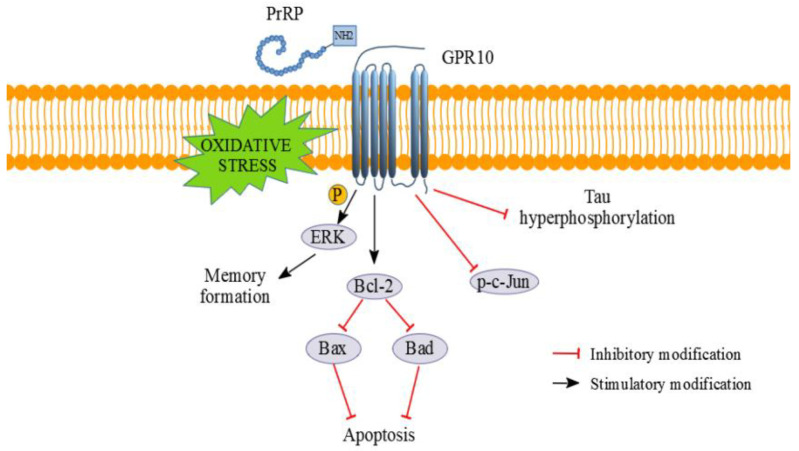
Schematic view of signaling pathways involved in PrRP neuroprotective and anti-apoptotic effect; P–phosphorylation.

To explain the anti-apoptotic and neuroprotective effects of PrRP on SH-SY5Y cells (Figure 10), we searched for changes in proteins of the Bcl-2 protein family, whose members act as anti- or pro-apoptotic agents. The apoptotic activator Bcl-2-associated X protein (Bax) was increased in the MG-treated cells and attenuated by PrRP31 and palm^11^-PrRP31, similar to our and other studies [27,58], and the prosurvival protein Bcl-2 was decreased in the MG-treated cells and was increased by PrRP31 and palm^11^-PrRP31. Bcl-2 directly binds to Bax, forming a nonactive heterodimer and blocking the formation of the active pro-apoptotic Bax homodimer. The ratio of Bax/Bcl-2, regarded as an apoptosis marker, suggests decreased apoptosis in the cells treated with PrRP31 and palm^11^-PrRP31 [59]. MG induced the phosphorylation of the pro-apoptotic protein Bcl-2-associated death promoter (Bad), expressed at higher levels in cancer cells was decreased by both PrRP and palm^11^-PrRP31 [60].

c-Jun is a component of the transcription factor activator protein-1 (AP-1). The transcriptional activity of c-Jun is regulated by phosphorylation at Ser63 and Ser73 through stress-activated protein kinases/Jun amino-terminal kinase (SAPK/JNK) [61]. Similarly, as in the study of Du [62], p-c-Jun (Ser73) was increased after incubation with MG and was reduced by pretreatment with PrRP31 and palm^11^-PrRP31. In this study, AP-1-regulated genes are involved in diverse biological functions, including cell proliferation, differentiation, and apoptosis, as well as transformation, invasion, and metastasis, depending on cell type and context [63,64]. Decreased activation of ERK can play an important role in the development of neurodegenerative diseases [51]. Our results showed that MG similarly attenuated ERK activation analogously to the study of Heimfarth [65], but this parameter was increased by PrRP31 and palm^11^-PrRP31 in this study.

In accordance with a previous study [28], we confirmed MG-induced Tau hyperphosphorylation at Ser396 and at Ser198/199/202 (Tau 1 antibody) in SH-SY5Y cells as in [28], and this change was attenuated by both PrRP31 and palm^11^-PrRP31 in this study. Furthermore, decreased Tau phosphorylation after palm^11^-PrRP31 was consistent with our previous in vivo study, which showed that palm^11^-PrRP31 inhibited the phosphorylation of Tau (Ser396/Ser404) in THY-Tau22 mice overexpressing mutated human Tau [17], and with our previous in vitro study, which showed that palm^11^-PrRP31 attenuated hypothermia-induced phosphorylation of Tau (Ser396) in SH-SY5Y cells [66].

In conclusion, PrRP and its lipidized analog increased viability in the MG-stressed SH-SY5Y cells and primary cortical cells. PrRP and its lipidized analog decreased the Bax/Bcl-2 ratio and activation of pro-apoptotic Bad protein and p-c-Jun (Ser73), suggesting decreased apoptosis in the cells. PrRP and its lipidized analog increased ERK activation, promoting cell survival and growth [50]. Furthermore, Tau hyperphosphorylation at Ser396 and at Ser198/199/202 was decreased after palm^11^-PrRP31 confirming our previous in vivo study [17] and supporting the idea of its potential neuroprotectivity.

## 4. Materials and Methods

### 4.1. Peptide Synthesis

Human PrRP31 and its lipidized analog palm^11^-PrRP31 (see Table 1 for structures) were synthesized and purified using the Fmoc strategy at the Institute of Organic Chemistry and Biochemistry, Academy of Sciences of the Czech Republic (IOCB AS CR), as described previously by [67,68]. The scrambled-palm^11^-PrRP31 analog (scrambled) used in this study as a negative control was derived from the structure of palm^11^-PrRP31, where arginines were substituted with citrullines (Cit) and the sequence of PrRP31 was randomly scrambled (Table 1). All peptides were dissolved in deionized water. Human insulin, used in the study as a positive control, was a gift from the group of Dr Jiří Jiráček, IOCB AS CR, Prague.

### 4.2. Chemicals

MTT (3-[4,5-dimethylthiazol-2-yl]-2,5-diphenyl tetrazolium bromide; thiazolyl blue), methylglyoxal solution (40% in H_2_O), and other common chemicals were obtained from Sigma–Aldrich (St. Louis, MO, USA). Rapamycin (m-Tor inhibitor), SB216736 (GSK-3β inhibitor), and U0126 (ERK inhibitor) were purchased from Tocris, Bristol, UK.

### 4.3. Cell Lines

The SH-SY5Y (ATCC^®^ CRL-2266™) neuroblastoma cell line, obtained from LGC standards (Teddington, London, UK), was grown in DMEM supplemented with 10% heat-inactivated fetal bovine serum, 1% nonessential amino acids, 1% streptomycin/penicillin, and 2 mM L-glutamine at 37 °C in a 5% CO_2_ humidified incubator. The medium was changed every 3–4 days, and the cells were subcultured as required.

Primary rat cortical neurons (A10840-02) obtained from Thermo Fisher Scientific (Waltham, MA, USA,) were grown in neurobasal medium supplemented with 2% B27, 200 mM L-glutamine and 1% antibiotic-antimycotic agent. Cells were seeded at 80,000 cells/well in 96-well plates coated with poly-D-lysine (0.1 mg/mL) and laminin (20 µg/mL), and the medium was changed every 3-4 days. The plates were maintained at 37 °C in a 5% CO_2_ humidified incubator for 10 days.

### 4.4. Differentiation of SH-SY5Y Cells

The SH-SY5Y cell line was differentiated into cells with morphological and biochemical characteristics of mature neurons using retinoic acid, which is described as a preferable model for neuroscience [69]. SH-SY5Y cells were grown in DMEM supplemented with 10% heat-inactivated fetal bovine serum, 1% nonessential amino acids, 1% streptomycin/penicillin, and 2 mM L-glutamine, enriched with 0.05 mM retinoic acid at 37 °C in a 5% CO_2_ humidified incubator. Cells were seeded at 50,000 cells/well in 24-well plates and differentiated for two weeks, and the medium was changed every 3−4 days. The saturable specific binding of ^125^I-PrRP31 to differentiated SH-SY5Y cells entailed decreased specific binding, resulting in an increase in Ki in the competitive binding of ^125^I-PrRP31 to SH-SY5Y cells. Therefore, differentiation was not suitable for our study.

### 4.5. Cell Viability Measurement

The MTT test was used to measure cell viability. The MTT reagent was dissolved in RPMI-1640 without phenol red. Cells were incubated for 2 h with a 10% solution of MTT at 37 °C. Living cells converted soluble MTT to insoluble formazan, which was subsequently dissolved in DMSO. The absorbance was measured at a wavelength of 560 nm.

For this assay, cells were cultured in 96-well plates at a density of 40,000 cells per well. The growth medium was exchanged for serum-free DMEM 16 h before the experiment. First, the cells were pretreated with 1 × 10^−5^ M or 1 × 10^−7^ M PrRP31 or palm^11^-PrRP31 (in octuplicates) for 4 h to prevent MG cytotoxic effects; then, MG was added to a final concentration of 0.6 mM or 1.2 mM. The cells were incubated at 37 °C for 16 h. Second, after exposure to MG, cells were first incubated for 16 h at 37 °C with MG at a final concentration of 0.6 mM or 1.2 mM and subsequently treated with PrRP31 or palm^11^-PrRP31 at a concentration of 1 × 10^−5^ M or 1 × 10^−7^ M PrRP31 or palm^11^-PrRP31 for 4 h (in octuplicates). At the end of the experiment, the MTT test was carried out.

### 4.6. Western Blotting

For the investigation of the molecular mechanism of PrRP31 and its analog in the cells, signaling pathways were studied by Western blotting (WB). The cells were cultured in 6-well plates at a density of 800,000 cells per well. Growth medium was exchanged for serum-free DMEM 16 h before an experiment, and then, the cells were incubated with PrRP31, palm^11^-PrRP31, or scrambled peptide at a final concentration of 1 × 10^−5^ M or insulin at a concentration of 1 × 10^−7^ M at 37 °C for 8 min. The cells were rapidly cooled on ice and lysed in 400 µl of Laemmli sample buffer (62.5 mM Tris-HCl with pH 6.8, 2% SDS, 10% glycerol, 0.01% bromophenol blue, 5% mercaptoethanol, 50 mM NaF, and 1 mM Na_3_VO_4_) and stored at −20 °C. Afterward, the cell lysates were analyzed by WB. SDS-electrophoresis and WB were carried out using a Criterion system with a 26-well 4−15% Criterion^TM^ TGX^TM^ Precast Midi Protein Gel (Bio-Rad, Hercules, CA, USA) at a constant voltage of 200 V or 100 V, respectively. Nitrocellulose membranes were blocked in 5% nonfat milk or 5% BSA at room temperature for 1 h, as shown in Table 2.

Subsequently, the membranes were incubated overnight with primary antibody (Ab) at 4 °C, according to the manufacturer´s instructions (Table 2). The list of antibodies used and their appropriate dilutions in TBS/tween-20 are shown in Table 2. The next day, the membranes were incubated with an HRP-linked secondary antibody for 1 h at room temperature. Chemiluminescence was visualized with a ChemiDoc^TM^ System (Bio-Rad). Band intensities were normalized using glyceraldehyde 3-phosphate dehydrogenase (GAPDH) as an internal loading control.

Eight minutes of incubation was performed in a series of experiments with different exposure times (5, 10, 15, 30, and 60 min) to PrRP31 and its palmitoylated analog. Activation of analogs was strongest after 10 min. Then, the cells were treated with inhibitors of signaling pathways for 2 min, and analogs were added for 8 min. The time of exposure was set to 8 min to ensure that the incubation time for PrRP31 and its palmitoylated analog was the same. The controls and compounds were tested in triplicate in three independent experiments. The PI3K/Akt pathway is one of the main signaling pathways of insulin; therefore, insulin was used as a positive control. Scrambled peptide was used as a negative control. Activation is expressed as the ratio of the activated protein to the total amount of the protein. GAPDH was used as a loading control.

For further research, inhibitors of signaling pathways were used. The growth medium was exchanged for serum-free DMEM 16 h before the experiment. On the day of the experiment, cells were incubated for 2 min with rapamycin, SB216736 or U0126 at a final concentration of 1 × 10^−6^ M; then, PrRP31, palm^11^-PrRP31 or scrambled peptide at a final concentration of 1 × 10^−5^ M or insulin at a concentration of 1 × 10^−7^ M was added for 8 min. The plates were stored at 37 °C. The controls and compounds were tested in triplicate.

For analysis of the molecular mechanism during MG stress, the growth medium was exchanged for serum-free DMEM 16 h before the experiment. The cells were pretreated with 1 × 10^−5^ M PrRP31 or palm^11^-PrRP31 for 4 h; then, MG was added to a final concentration of 0.6 mM, and the cells were incubated at 37 °C for 16 h. The controls and compounds were tested in triplicate.

### 4.7. Analysis of Data and Statistics

Data are presented as the mean ± SEM and were analyzed with GraphPad Software (San Diego, CA, USA). Saturation binding curves were plotted to compare the best fit for single binding site models (K_d_, B_max_, and IC_50_ values were obtained from nonlinear regression analysis). Data from cell viability measurements and WB were analyzed using one-way ANOVA, followed by Dunnett´s post hoc test or Student´s *t*-test, as stated in the Figures. *p* < 0.05 was considered statistically significant.

## 5. Conclusions

PrRP31 and its lipidized analog palm^11^-PrRP31 were proven to activate cellular signaling pathways linked to proper memory formation, such as ERK1/2, or kinases implicated in insulin signaling pathways, such as Akt, GSK-3β, and mTOR, which are important in the potential neuroprotective properties of PrRP. Moreover, in MG-stressed SH-SY5Y cells, treatment with PrRP31 or its lipidized analog led to increased cell viability and suppression of apoptosis. The results showed the potential neuroprotective effect of PrRP31 and palm^11^-PrRP31, suggesting their potential for the treatment of neurodegenerative diseases.

## Figures and Tables

**Table 1:** Structure of human prolactin-releasing peptide 31 (PrRP31) and its analogs.

Analogs	Sequence
PrRP31	SRTHRHSMEI**R^11^**TPDINPAWYASRGIRPVGRF-NH_2_
Palm^11^-PrRP31	SRTHRHSMEI**K^11^** (γ-E (N-palm))TPDINPAWYASRGIRPVGRF-NH_2_
Scrambled-palm^11^-PrRP31	GHFTHSIRMI **K^11^** (γ-E (N-palm))TPRNASVYARPCitDWWGICitPES

Cit-citrullin.

**Table 2:** List of primary antibodies used for immunoblotting and their dilutions.

Antibody	Blocking	Dilution for Western Blot	Provider
Akt rabbit Ab	5% milk	1:1000, 5% BSA	Cell signaling Technology, Beverly MA, USA
p-Akt (Ser473) rabbit Ab	5% milk	1:1000, 5% BSA	Cell signaling Technology, Beverly MA, USA
p-Akt (Thr308) rabbit Ab	5% milk	1:1000, 5% BSA	Cell signaling Technology, Beverly MA, USA
Bad rabbit Ab	5% milk	1:1000, 5% BSA	Cell signaling Technology, Beverly MA, USA
p-Bad (Ser112) rabbit Ab	5% BSA	1:1000, 5% BSA	Cell signaling Technology, Beverly MA, USA
p-Bad (Ser136) rabbit Ab	5% BSA	1:1000, 5% BSA	Cell signaling Technology, Beverly MA, USA
Bax rabbit Ab	5% milk	1:1000, 5% BSA	Cell signaling Technology, Beverly MA, USA
Bcl2 rabbit Ab	5% BSA	1:1000, 5% BSA	Cell signaling Technology, Beverly MA, USA
CREB mouse Ab	5% milk	1:1000, 5% milk	Cell signaling Technology, Beverly MA, USA
p-CREB (Ser133) mouse Ab	5% milk	1:1000, 5% milk	Cell signaling Technology, Beverly MA, USA
ERK mouse Ab	5% milk	1:2000, 5% milk	Cell signaling Technology, Beverly MA, USA
p-ERK (Thr202/Tyr204) mouse Ab	5% milk	1:2000, 5% milk	Cell signaling Technology, Beverly MA, USA
GSK-3β rabbit Ab	5% milk	1:1000, 5% BSA	Cell signaling Technology, Beverly MA, USA
p-GSK-3β (Ser9) rabbit Ab	5% milk	1:1000, 5% BSA	Cell signaling Technology, Beverly MA, USA
GAPDH mouse Ab	5% milk	1:1000, 5% milk	Cell signaling Technology, Beverly MA, USA
c-Jun rabbit Ab	5% BSA	1:1000, 5% BSA	Cell signaling Technology, Beverly MA, USA
p-c-Jun (Ser73) rabbit Ab	5% BSA	1:1000, 5% BSA	Cell signaling Technology, Beverly MA, USA
m-Tor rabbit Ab	5% BSA	1:1000, 5% BSA	Cell signaling Technology, Beverly MA, USA
p-m-Tor (Ser2448) rabbit Ab	5% BSA	1:1000, 5% BSA	Cell signaling Technology, Beverly MA, USA
PDK1 rabbit Ab	5% milk	1:1000, 5% BSA	Cell signaling Technology, Beverly MA, USA
p-PDK1 (Ser241) rabbit AB	5% milk	1:1000, 5% BSA	Cell signaling Technology, Beverly MA, USA
PI3K p85 rabbit Ab	5% milk	1:1000, 5% BSA	Cell signaling Technology, Beverly MA, USA
Tau 1 mouse Ab	5% milk	1:10000, 5% milk	Merck Millipore, Burlington MA, USA
Tau 5 mouse Ab	5% milk	1:5000, 5% milk	Thermo Fisher Scientific Inc., Waltham, MA, USA
p-Tau (Ser396) rabbit Ab	5% BSA	1:10000, 5% BSA	Thermo Fisher Scientific Inc., Waltham, MA, USA

anti-rabbit IgG HRP-linked antibody and anti-mouse IgG horseradish peroxidase (HRP)-linked antibody were purchased from Cell Signaling Technology (Beverly, MA, USA).
